# Suberoylanilide Hydroxamic Acid Attenuates Autoimmune Arthritis by Suppressing Th17 Cells through NR1D1 Inhibition

**DOI:** 10.1155/2019/5648987

**Published:** 2019-10-24

**Authors:** Da Som Kim, Hong-Ki Min, Eun Kyung Kim, Seung Cheon Yang, Hyun Sik Na, Seon-Yeong Lee, Jeong-Won Choi, Kyung-Ah Jung, Seung-Ki Kwok, Sung-Hwan Park, Mi-La Cho

**Affiliations:** ^1^The Rheumatism Research Center, Catholic Research Institute of Medical Science, College of Medicine, The Catholic University of Korea, Seoul, Republic of Korea; ^2^Division of Rheumatology, Department of Internal Medicine, Seoul St. Mary's Hospital, College of Medicine, The Catholic University of Korea, Seoul, Republic of Korea; ^3^Impact Biotech, Seoul, Republic of Korea; ^4^Laboratory of Immune Network, Conversant Research Consortium in Immunologic Disease, College of Medicine, The Catholic University of Korea, Seoul, Republic of Korea; ^5^Department of Medical Lifescience, College of Medicine, The Catholic University of Korea, Seoul, Republic of Korea; ^6^Department of Biomedicine & Health Sciences, College of Medicine, The Catholic University of Korea, Seoul, Republic of Korea

## Abstract

Rheumatoid arthritis (RA) is a type of systemic autoimmune arthritis that causes joint inflammation and destruction. One of the pathological mechanisms of RA is known to involve histone acetylation. Although the histone deacetylase (HDAC) inhibitor suberoylanilide hydroxamic acid (SAHA) can attenuate arthritis in animal models of RA, the mechanism underlying this effect is poorly understood. This study was performed to examine whether SAHA has therapeutic potential in an animal model of RA and to investigate its mechanism of action. Collagen-induced arthritis (CIA) mice were orally administered SAHA daily for 8 weeks and examined for their arthritis score and incidence of arthritis. CD4^+^ T cell regulation following SAHA treatment was confirmed in splenocytes cultured under type 17 helper T (Th17) cell differentiation conditions. Clinical scores and the incidence of CIA were lower in mice in the SAHA treatment group compared to the controls. In addition, SAHA inhibited Th17 cell differentiation, as well as decreased expression of the Th17 cell-related transcription factors pSTAT3 Y705 and pSTAT3 S727. *In vitro* experiments showed that SAHA maintained regulatory T (Treg) cells but specifically reduced Th17 cells. The same results were obtained when mouse splenocytes were cultured under Treg cell differentiation conditions and then converted to Th17 cell differentiation conditions. In conclusion, SAHA was confirmed to specifically inhibit Th17 cell differentiation through nuclear receptor subfamily 1 group D member 1 (NR1D1), a factor associated with Th17 differentiation. The results of the present study suggested that SAHA can attenuate CIA development by inhibition of the Th17 population and maintenance of the Treg population through NR1D1 inhibition. Therefore, SAHA is a potential therapeutic candidate for RA.

## 1. Introduction

Rheumatoid arthritis (RA) is a systemic autoimmune arthritis characterized by synovitis and progressive destruction of the cartilage and bone [1]. Although the precise pathogenic mechanism remains unclear, T cells abundant in the synovial tissue play a central role in the pathogenesis orchestrating innate and adaptive immune responses [2]. Type 17 helper T (Th17) cells and their cytokines (e.g., interleukin- (IL-) 17, IL-21, and IL-22) contribute to the progression of inflammatory diseases, including RA [3]. IL-17, a cytokine mainly expressed by Th17 cells, is known to promote the activation of synoviocytes [4]. Regulatory T (Treg) cells are known to suppress autoimmunity and play an anti-inflammatory role in the pathogenesis of RA [5, 6]. Therefore, targeting Th17/Treg cells may be effective in the treatment of RA.

Several epigenetic modifications, such as DNA methylation and histone acetylation, have been shown to have pathological roles in RA progression [7]. In RA, fibroblast-like synoviocytes (FLSs) have been shown to exhibit abnormal histone acetylation, and tumor-necrosis factor (TNF) was shown to increase the level of histone deacetylase (HDAC) 1 expression [8]. Several previous studies have indicated therapeutic effects of HDAC inhibitors in RA [9, 10]. Suberoylanilide hydroxamic acid (SAHA) is a pan-HDAC inhibitor, which has been shown to have therapeutic effects in experimental autoimmune encephalomyelitis [11] and experimental autoimmune uveitis [12] mediated by regulation of CD4^+^ T cell subsets. SAHA has been shown to increase apoptosis of FLSs in RA patients mostly through NF-*κ*B [13, 14] and suppressed proinflammatory cytokines, proangiogenic factors, nitric oxide, and matrix metallopeptidase [14]. In addition, SAHA decreased production of chemotactic factors via the p38 mitogen-activated protein kinase pathway in RA FLS [15]. Arthritis has been shown to be alleviated by SAHA in a collagen-induced arthritis (CIA) animal model [16, 17], but the mechanism underlying this effect is not yet clear. Recently, sodium butyrate, a representative anti-inflammatory metabolite of gut microbiota, was shown to have an anti-inflammatory effect in a mouse model of RA by regulating osteoclastogenesis and Th17/Treg cell imbalance [18]. In a previous study, sodium butyrate was shown to regulate HDAC2 in osteoclasts and HDAC8 in CD4^+^ T cells and to eventually control transcriptional activity of estrogen-related receptor alpha (ERR*α*) and nuclear receptor subfamily 1 group D member 1 (NR1D1) [18].

The present study was performed to investigate whether SAHA has a therapeutic effect on RA through modulating Th17 and Treg cells in a mouse model of CIA. We also examined the underlying mechanism by which SAHA regulates the CD4^+^ T cell subset with regard to control of NR1D1.

## 2. Materials and Methods

### 2.1. Induction of Collagen-Induced Arthritis and Treatment with SAHA

CIA was generated in male DBA/1J mice (Orient Bio, Korea). Mice were maintained in a specific-pathogen-free environment. DBA/1J mice received intradermal injection of 100 *μ*g of chicken type II collagen (CII; Chondrex Inc., Redmond, WA, USA) mixed with complete or incomplete Freund's adjuvant (Chondrex Inc.). To assess the influence of SAHA on the severity of symptoms in the CIA model, mice were treated with 50 mg/kg SAHA or vehicle alone via daily oral feeding after booster immunization. Mice were sacrificed at 8 weeks after induction of CIA.

Mice were considered to have arthritis when significant changes in redness and/or swelling were noted in the digits or other parts of the paws. Knee joint inflammation was scored visually after dissection on a scale from 0 to 4 (0, noninflamed; 1, minimal; 2, mild; 3, moderate; and 4, severe inflammation).

All experimental procedures were approved by the Animal Research Ethics Committee at the Catholic University of Korea (approval number 2018-0115-02).

### 2.2. Collagen Type II-Specific Antibodies

Blood was obtained from the orbital sinus of CIA mice, and the serum levels of CII-specific mouse IgG, IgG1, and IgG2a were measured using enzyme-linked immunosorbent assay (ELISA) kits (Bethyl Laboratories, Montgomery, TX, USA).

### 2.3. Histological Analysis and Immunohistochemistry

Histological analysis was performed to determine the extent of joint damage. Sections were stained using hematoxylin and eosin (H&E) and safranin O. Joint tissue sections were stained with antibodies against inflammatory cytokines IL-17, IL-6, IL-1*β*, and TNF-*α*. The samples were visualized by microscopy (Olympus, Center Valley, PA, USA).

### 2.4. Micro-CT Imaging and Analysis

Micro-CT imaging and analysis was performed using a bench-top cone-beam type in vivo animal scanner (SKYSCAN1172 micro-CT, Bruker Micro-CT, Belgium). The hind paws of the dead mice were dissected and immediately fixed in 10% neutral buffered formalin. Samples were imaged with settings of 60 kVp and 169 *μ*A and an aluminum 0.5 mm thick filter. The pixel size was 11.88 *μ*m, and the rotation step was 0.7°. The cross-sectional images were reconstructed using a filtered back-projection algorithm (NRecon software, Bruker Micro-CT, Belgium).

### 2.5. Confocal Microscopy

Total splenocytes of normal C57BL/6 mice were placed in the appropriate wells of a cytospin chamber (Thermo Fisher Scientific, Kalamazoo, MI, USA) and centrifuged at 700 × *g* for 3 min. Spleen tissue cryosections (7 *μ*m thick) were fixed with methanol-acetone and stained with FITC-, PE-, PerCP-Cy5.5-, or APC-conjugated monoclonal antibodies against mouse CD4, IL-17, CD25, Foxp3, DAPI, NR1D1, pSTAT3 S727, and pSTAT3 Y705 (eBioscience, San Diego, CA, USA). After overnight incubation at 4°C, the stained samples were visualized by confocal microscopy (LSM 510 Meta; Zeiss, Oberkochen, Germany).

### 2.6. Mouse *In Vitro* Experiments

To establish Th17 cell-polarizing conditions, splenocytes were isolated from normal C57BL/6 mice and the cells were cultured for 3 days. The cells were stimulated with anti-CD3 (0.5 *μ*g/mL), anti-CD28 (1 *μ*g/mL), anti-interferon-*γ* (IFN-*γ*) (5 *μ*g/mL), anti-IL-4 (5 *μ*g/mL), and IL-6 (20 ng/mL) antibodies and transforming growth factor-*β* (TGF-*β*) (2 ng/mL). Recombinant mouse IL-6 and antibodies against IFN-*γ* and IL-4 were purchased from R&D Systems (Minneapolis, MN, USA), and TGF-*β* was purchased from PeproTech (Rocky Hill, NJ, USA).

To convert Treg cells to Th17 cells, splenocytes from normal C57BL/6 mice were cultured for 3 days under Treg cell-polarizing conditions and cultured for 3 days in fresh media under Th17-polarizing conditions. To establish Treg cell-polarizing conditions, splenocytes were stimulated with anti-CD3 (0.5 *μ*g/mL), anti-CD28 (1 *μ*g/mL), anti-IFN-*γ* (5 *μ*g/mL), anti-IL-4 (5 *μ*g/mL), and TGF-*β* (5 ng/mL) antibodies. The Th17 cell-polarizing conditions were the same as described above. To compare the effects of SAHA on Th17 differentiation with regard to NR1D1, SR8278, an antagonist of NR1D1, was used under Th17-polarizing conditions.

### 2.7. Real-Time Polymerase Chain Reaction

Messenger RNA (mRNA) was extracted using TRI Reagent (Molecular Research Center, Inc., Cincinnati, OH, USA). Complementary DNA (cDNA) was synthesized using a SuperScript Reverse Transcriptase system (TaKaRa, Shiga, Japan). A LightCycler 2.0 instrument (software version 4.0; Roche Diagnostics, Penzberg, Germany) was used for amplification by polymerase chain reaction (PCR). All reactions were performed using the LightCycler FastStart DNA Master SYBR Green I mix (TaKaRa). The following primers were used: IL-17, 5′-CCT-CAA-AGC-TCA-GCG-TGT-CC-3′ (sense) and 5′-GAG-CTC-ACT-TTT-GCG-CCA-AG-3′ (antisense); IL-10, 5′-GGC-CCA-GAA-ATC-AAG-GAG-CA-3′ (sense) and 5′-AGA-AAT-CGA-TGA-CAG-CGC-CT-3′ (antisense); NR1D1, 5′-GCC-ATG-TTT-GAC-TTC-AGC-G-3′ (sense) and 5′-AAT-TCT-CCA-TTC-CCG-AGC-G-3′ (antisense); and *β*-actin, 5′-GAA-ATC-GTG-CGT-GAC-ATC-AAA-G-3′ (sense) and 5′-TGT-AGT-TTC-ATG-GAT-GCC-ACA-G-3′ (antisense). All mRNA levels were normalized relative to that of *β*-actin as an internal control.

### 2.8. Flow Cytometry

For intracellular staining, cells were restimulated with 25 ng/mL phorbol 12-myristate 13-acetate and 250 ng/mL ionomycin (both from Sigma-Aldrich) for 4 h in the presence of GolgiStop (BD Biosciences, Sparks, MD, USA). Murine splenocytes were stained with surface PerCP-conjugated anti-CD4 (eBioscience) and APC-conjugated anti-CD25 (BioLegend, San Diego, CA, USA) antibodies. After fixation and permeabilization, cells were stained with FITC-conjugated anti-IL-17 or PE-conjugated anti-Foxp3 antibodies (eBioscience). Events were collected and analyzed with FlowJo software (Tree Star, Ashland, OR, USA).

### 2.9. Statistical Analysis

All statistical analyses were performed using GraphPad Prism (v.5 for Windows; GraphPad Software, Inc., La Jolla, CA, USA). Data are presented as means ± standard deviation (SD). Comparisons of the numerical data obtained from two groups were performed by Student's *t*-test or the Mann-Whitney *U*-test. Differences in the mean values of various groups were subjected to analysis of variance (ANOVA) with a *post hoc* test. *p* values less than 0.05 (two-tailed) were considered indicative of statistical significance.

## 3. Results

### 3.1. Antiarthritic Efficacy of SAHA in CIA Mice

**Figure 1:**
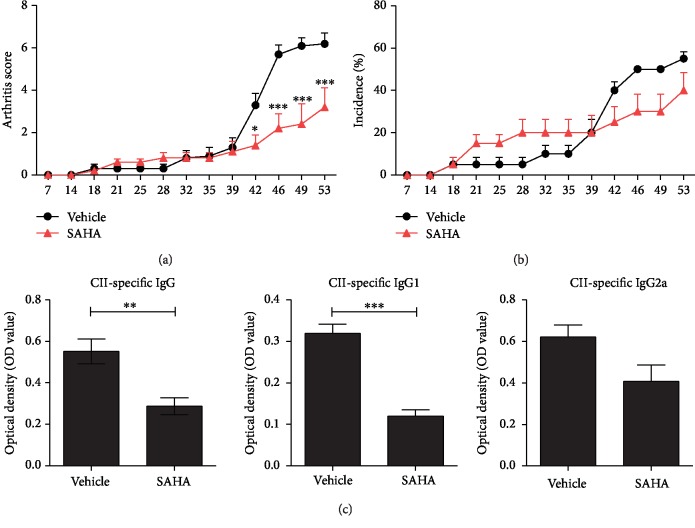
Antiarthritic efficacy of SAHA in CIA mice. (a, b) CIA mice were treated with 50 mg/kg SAHA or vehicle alone via daily oral feeding after secondary immunization. The arthritis score and incidence are presented (*n* = 5). (c) The serum concentrations of CII-specific IgG, IgG1, and IgG2a were evaluated. ^∗^*p* < 0.05, ^∗∗^*p* < 0.01, and ^∗∗∗^*p* < 0.001.

To determine whether SAHA has therapeutic efficacy in the RA mouse model, we treated CIA-induced DBA/1J mice with SAHA. The arthritis score and incidence rate were lower in the SAHA treatment group than in the negative controls (Figures 1(a) and 1(b)). Serum CΙΙ-specific IgG, IgG1, and IgG2a levels were reduced by the administration of SAHA (Figure 1(c)). These results suggested that SAHA alleviates arthritis.

### 3.2. Effects of SAHA on Joint Inflammation in CIA Mice

**Figure 2:**
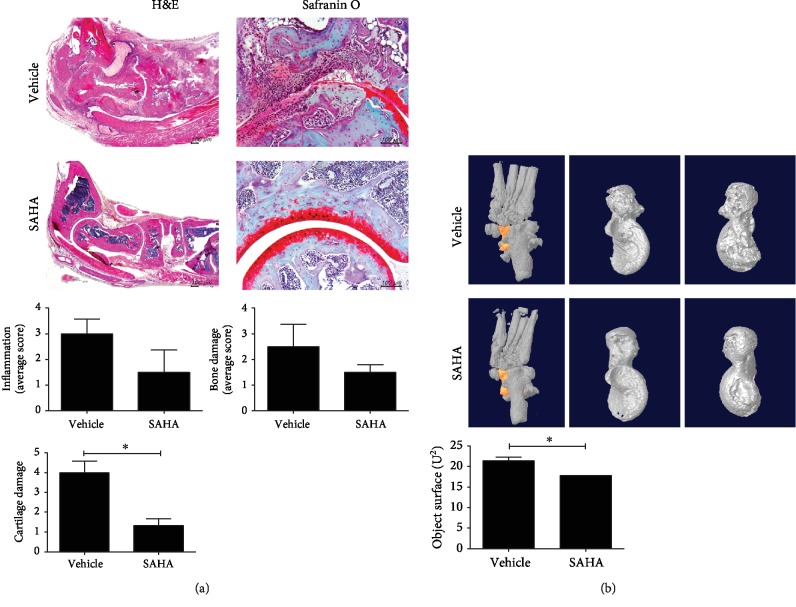
Effects of SAHA on joint inflammation in CIA mice. (a) Histological analysis of joint tissue sections with H&E (original magnification, 40x) and safranin O (original magnification, 200x). Joint inflammation and damage scores are shown (*n* = 3). (b) Representative micro-CT appearances of the joints at 8 weeks after CIA induction. The position of the talus in the joint and a representative picture of the talus in two directions. Objective surface of micro-CT images from joints is shown (*n* = 3). ^∗^*p* < 0.05.

Tissue staining and microcomputed tomography (CT) analyses were performed to determine whether SAHA could regulate joint inflammation. Staining with H&E and safranin O showed that SAHA tended to decrease joint inflammation and alleviate the degree of bone and cartilage damage in the joint tissues of CIA mice (Figure 2(a)). In addition, micro-CT confirmed that SAHA reduced the objective surface of the joint (Figure 2(b)). The micro-CT analyses confirmed the therapeutic effect of SAHA in this CIA mouse model.

### 3.3. Influence of SAHA on Inflammatory Cytokines in the Joints of CIA Mice

**Figure 3:**
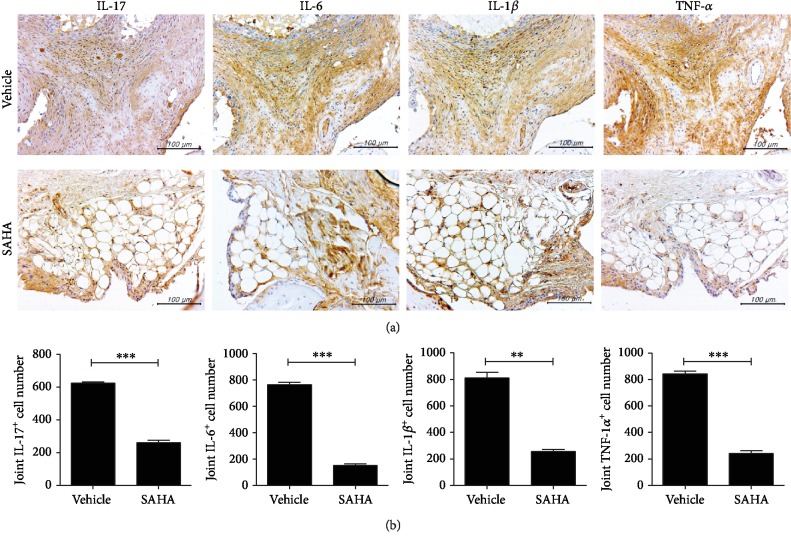
Influence of SAHA on inflammatory cytokines in joints in CIA mice. (a) Histological features of the joints of CIA mice (*n* = 3). Immunohistochemical staining for IL-17, IL-6, IL-1*β*, and TNF-*α* (original magnification, 400x). (b) Quantitative results of immunohistochemical staining are shown. ^∗∗^*p* < 0.01 and ^∗∗∗^*p* < 0.001.

We examined whether SAHA regulates inflammatory cytokines in the joints. The expression levels of IL-17, IL-6, IL-1*β*, and TNF-*α* in joint tissues were markedly reduced following treatment with SAHA (Figure 3(a)). The results of the staining were quantified for each proinflammatory cytokine (IL-17, IL-6, IL-1*β*, and TNF-*α*) staining cell count and consistently showed reduced cell counts in the SAHA treatment group (Figure 3(b)).

### 3.4. Effects of SAHA on T Cells in *Ex Vivo* Spleen Tissue

**Figure 4:**
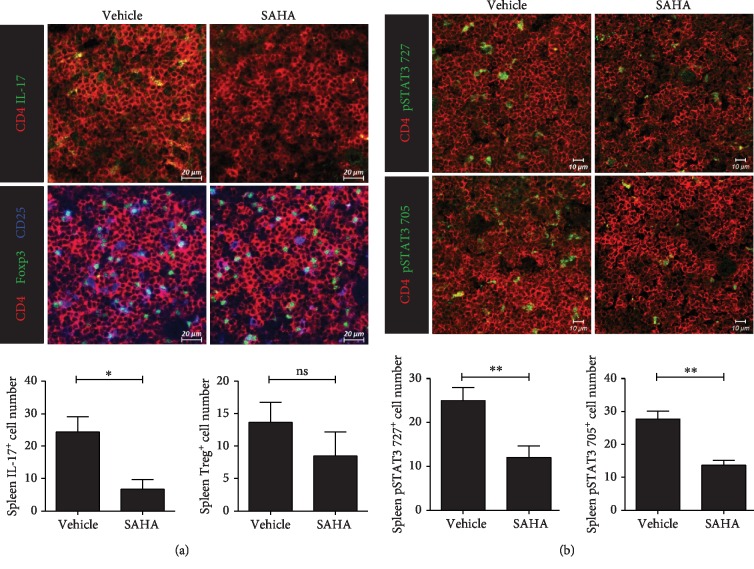
Effects of SAHA on T cells in *ex vivo* spleen tissue. (a) Confocal microscopy of spleen cryosections, stained for Th17 or Treg cells. The numbers of positive cells are shown (*n* = 3). (b) Confocal microscopy of spleen cryosections, stained for pSTAT3 727 or pSTAT3 705 (original magnification, 400x). The numbers of positive cells are shown (*n* = 3). ^∗^*p* < 0.05 and ^∗∗^*p* < 0.01.

Confocal staining was performed to determine whether SAHA regulates Th17 and Treg cells in spleen tissues. As shown in Figure 4(a), Th17 cells were significantly decreased and Treg cells were maintained in the SAHA treatment group. Tyr705- and Ser727-phosphorylated STAT3 was significantly inhibited by SAHA in the spleen tissues of CIA mice (Figure 4(b)). These observations indicated that SAHA effectively decreased Th17 cell counts, while Treg cells were maintained.

### 3.5. Effects of SAHA on T Cells *In Vitro* under Th17 Differentiation Conditions

**Figure 5:**
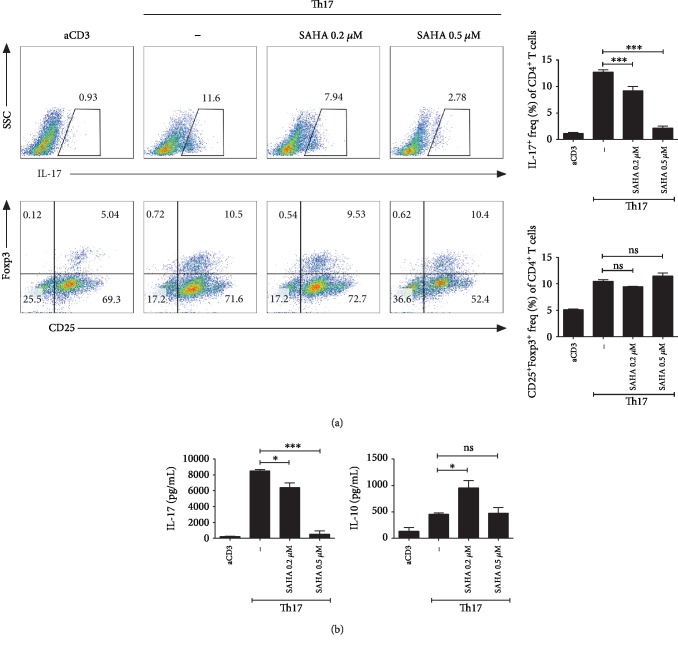
Effects of SAHA on T cells cultured *in vitro* under Th17 cell differentiation conditions. (a) Total splenocytes were extracted from the spleens of C57BL/6 mice and cultured with SAHA under Th17-polarizing conditions for 3 days. The cells were subjected to flow cytometric analysis of Th17 or Treg cells. Representative results are shown. (b) Three days after inducing Th17 cells, the protein levels of IL-17 and IL-10 were measured. ^∗^*p* < 0.05 and ^∗∗∗^*p* < 0.001.

We investigated the effects of SAHA on Th17 and Treg cells *in vitro*. Th17 cell numbers were reduced, while Treg cells were maintained when cells cultured under Th17 cell differentiation conditions were treated with SAHA at various concentrations (Figure 5(a)). In addition, IL-17 and IL-10 expression was confirmed in the supernatants of these cells (Figure 5(b)). These observations confirmed that the level of IL-17 was decreased by SAHA in a concentration-dependent manner, while SAHA showed no effect on IL-10 at any concentration examined.

### 3.6. Effects of SAHA on T Cells *In Vitro* under Treg to Th17 Cell Differentiation Conditions

**Figure 6:**
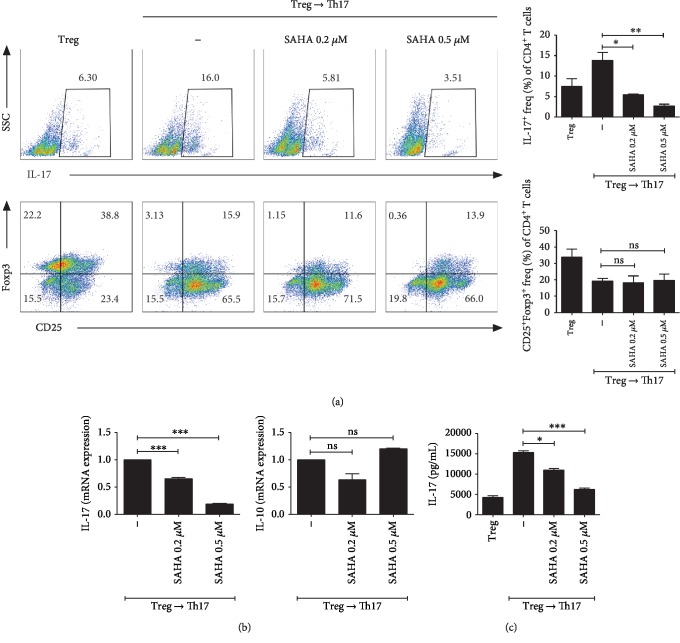
Effects of SAHA on T cells cultured *in vitro* under Treg to Th17 cell differentiation conditions. (a) Total splenocytes were extracted from the spleens of C57BL/6 mice and cultured under Treg-polarizing conditions for 3 days. The cells were converted to Th17-polarizing conditions with SAHA in fresh medium. The cells were subjected to flow cytometric analysis of Th17 or Treg cells. Representative results are shown. (b) Two days after inducing Th17 cells, the mRNA levels of IL-17 and IL-10 were measured. (c) The cell supernatants were examined for IL-17 by ELISA. ^∗^*p* < 0.05, ^∗∗^*p* < 0.01, and ^∗∗∗^*p* < 0.001.

To confirm that SAHA inhibits Th17 cells while maintaining Treg, we cultured splenocytes under Treg to Th17 differentiation conditions in the presence of various SAHA concentrations. Th17 cells were decreased and Treg cells were maintained by SAHA treatment (Figure 6(a)). In addition, IL-17 and IL-10 mRNA levels were significantly reduced and maintained by SAHA, respectively (Figure 6(b)). IL-17 cytokine expression was also reduced by SAHA in a dose-dependent manner (Figure 6(c)).

### 3.7. Target Molecule of SAHA

**Figure 7:**
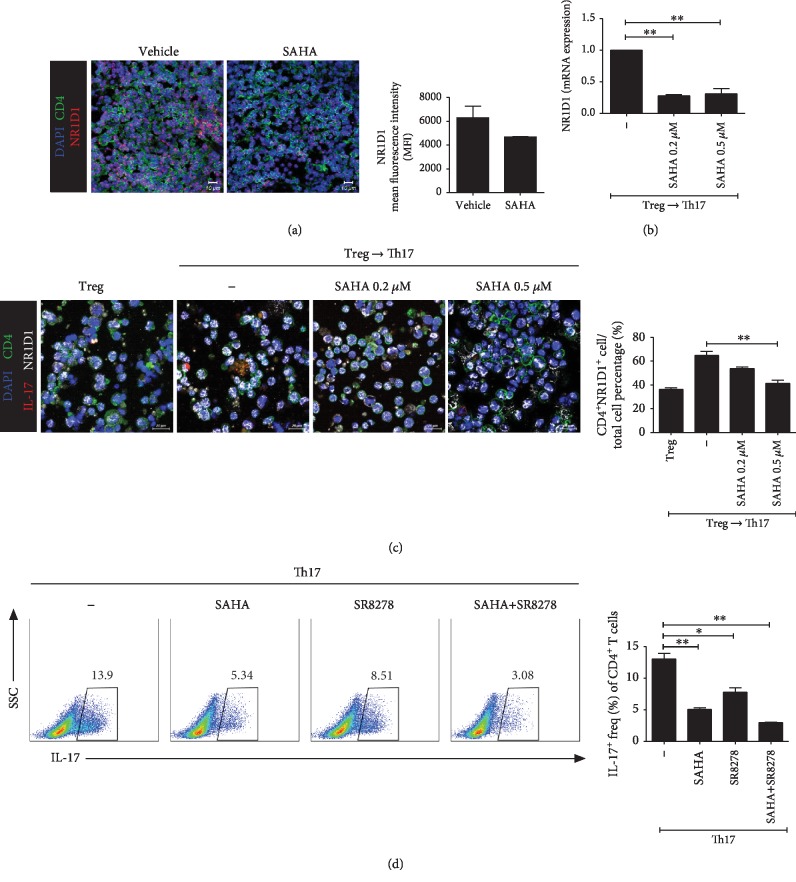
Target molecule of SAHA. (a) Confocal microscopy of spleen cryosections, stained for DAPI, CD4, and NR1D1 (original magnification, 400x). The mean fluorescence intensities are shown (*n* = 3). (b) Total splenocytes were cultured under Treg-polarizing conditions for 3 days. The cells were converted to Th17-polarizing conditions with SAHA for 2 days. NR1D1 mRNA expression was analyzed. (c) Total splenocytes were cultured under Treg-polarizing conditions for 3 days. The cells were converted to Th17-polarizing conditions with SAHA for 3 days. NR1D1 expression was confirmed in cells cultured for a total of 6 days by confocal microscopy. Relative bar charts are shown. (d) Total splenocytes were isolated from splenocytes and cultured with SAHA or SR8278 under Th17-polarizing conditions for 3 days, and Th17 cell populations were analyzed by flow cytometry. ^∗^*p* < 0.05, ^∗∗^*p* < 0.01, and ^∗∗∗^*p* < 0.001.

Confocal staining was performed in the spleens of CIA mice to determine whether SAHA could inhibit specific Th17 cell-related target molecules. As shown in Figure 7(a), NR1D1 expression levels were reduced by SAHA in the spleens of CIA mice. The NR1D1 mRNA level was reduced by SAHA in cells differentiated under Treg to Th17 cell differentiation conditions (Figure 7(b)). The number of cells expressing NR1D1 was increased under Treg to Th17 cell differentiation conditions compared to Treg differentiation conditions. The increased NR1D1 level was reduced by SAHA (Figure 7(c)). Th17 differentiation was suppressed in the 0.2 *μ*M SAHA, 5 *μ*M SR8278, and SAHA+SR8278 combination groups (Figure 7(d)). SAHA and SR8278 showed synergistic suppressive effects on Th17 differentiation (Figure 7(d)).

## 4. Discussion

The results of the present study demonstrated a preventive effect of SAHA on the development of autoimmune arthritis in a CIA mouse model. Although previous studies showed a suppressive effect of SAHA on RA, they did not demonstrate the underlying mechanism, especially at the transcriptional level in CD4^+^ T cells [13, 16]. We determined the mechanism by which SAHA, a pan-HDAC inhibitor, suppressed autoimmune arthritis with regard to regulating CD4^+^ T cell subsets as well as at the transcriptional level in CD4^+^ T cell differentiation.

There are several subsets of CD4^+^ T cells, i.e., Th1, Th2, Th17, and Treg cells [19]. Imbalance between Th17 and Treg populations is important in autoimmune-related diseases, and decreases in Th17 number and increases in Treg number have emerged as treatment targets [20]. The peripheral blood of RA patients shows an imbalance between Th17 and Treg cells [21, 22], and the number of Th17 cells was strongly correlated with disease activity in RA patients [23]. Furthermore, regulating Th17/Treg imbalance was shown to have a beneficial effect in a mouse model of RA [24, 25]. The results of the present study showed that the antiarthritic effect of SAHA was mediated by reduction of the Th17 count and maintenance of the Treg population.

SAHA is a pan-HDAC inhibitor that has been reported to arrest cells in cancer [26, 27] and to have therapeutic effects in several types of cancer [26, 27]. FLSs represent the major component of the pannus in RA and show massive proliferation and an aggressive phenotype, eventually resulting in bone and cartilage destruction. The proliferative properties of FLSs are similar to those of tumor cells, and FLSs in RA show alterations in several genetic and epigenetic processes [28]. HDAC inhibitors have been studied as a treatment option for autoimmune arthritis and have shown favorable results in some animal models of RA and peripheral blood mononuclear cells from RA patients [9, 10, 16, 17]. SAHA has been reported to directly decrease Th17 cells in experimental autoimmune encephalomyelitis [11] and experimental autoimmune uveitis [12] and reduces Th17 cell-inducing cytokines [29]. Although SAHA has been reported to inhibit the development of arthritis in animal models of RA, the mechanisms underlying these effects have not been elucidated [16, 17]. In the present study, we demonstrated the downregulation of Th17 and maintenance of Treg cells in CIA mice and showed that the underlying mechanism of Th17/Treg regulation by SAHA involves suppression of NR1D1.

Intrinsic cellular circadian clocks are known to be involved in the differentiation of CD4^+^ T cells [30]. NR1D1, one of the genes involved in the circadian clock mechanism, is critical for Th17 differentiation [31]. A recent study showed that sodium butyrate suppressed Th17 cell differentiation in a mouse model of RA via NR1D1 inhibition [18]. In the present study, we demonstrated similar results of SAHA, which was shown to suppress Th17 by controlling NR1D1. Further studies are needed to confirm the beneficial effect of SAHA observed here and to determine whether SAHA exerts similar beneficial effects in RA patients.

Treg cells are known to play a suppressive role in autoimmune diseases. However, these cells have plasticity depending on the extracellular environment and can change their phenotype [32]. In the present study, maintenance of Treg cells by SAHA was confirmed *in vitro*. Splenocytes in culture were switched from Treg-polarizing conditions to Th17 polarizing conditions, and maintenance of the Treg cell population was consistently observed.

## 5. Conclusions

This study confirmed that SAHA may have therapeutic efficacy in RA through regulation of Th17/Treg imbalance. Th17 cells are specifically targeted to control immune regulation by regulating the gene underlying the circadian clock mechanism, which is important for maintenance of homeostasis in the human body. SAHA is a novel therapeutic candidate for RA treatment.

## Data Availability

The data used to support the findings are available from the corresponding authors upon request.

## Abbreviations

RA: Rheumatoid arthritis
SAHA: Suberoylanilide hydroxamic acid
HDAC: Histone deacetylase
CIA: Collagen-induced arthritis
NR1D1: Nuclear receptor subfamily 1 group D member 1
CII: Chicken type II collagen
ELISA: Enzyme-linked immunosorbent assay
H&E: Hematoxylin and eosin
IFN-*γ*: Interferon-*γ*
TGF-*β*: Transforming growth factor-*β*
mRNA: Messenger RNA
cDNA: Complementary DNA
STAT3: Signal transducer and activator of transcription 3.
